# Synthesising Facial Macro- and Micro-Expressions Using Reference Guided Style Transfer

**DOI:** 10.3390/jimaging7080142

**Published:** 2021-08-11

**Authors:** Chuin Hong Yap, Ryan Cunningham, Adrian K. Davison, Moi Hoon Yap

**Affiliations:** 1 Department of Computing and Mathematics, Manchester Metropolitan University, Manchester M15 6BH, UK; Ryan.Cunningham@mmu.ac.uk (R.C.); M.Yap@mmu.ac.uk (M.H.Y.); 2 Faculty of Biology, Medicine and Health, The University of Manchester, Manchester M13 9PL, UK; adrian.davison@manchester.ac.uk

**Keywords:** micro-expressions, facial expressions, style transfer, generative adversarial network, facial action units

## Abstract

Long video datasets of facial macro- and micro-expressions remains in strong demand with the current dominance of data-hungry deep learning methods. There are limited methods of generating long videos which contain micro-expressions. Moreover, there is a lack of performance metrics to quantify the generated data. To address the research gaps, we introduce a new approach to generate synthetic long videos and recommend assessment methods to inspect dataset quality. For synthetic long video generation, we use the state-of-the-art generative adversarial network style transfer method—StarGANv2. Using StarGANv2 pre-trained on the CelebA dataset, we transfer the style of a reference image from SAMM long videos (a facial micro- and macro-expression long video dataset) onto a source image of the FFHQ dataset to generate a synthetic dataset (SAMM-SYNTH). We evaluate SAMM-SYNTH by conducting an analysis based on the facial action units detected by OpenFace. For quantitative measurement, our findings show high correlation on two Action Units (AUs), i.e., AU12 and AU6, of the original and synthetic data with a Pearson’s correlation of 0.74 and 0.72, respectively. This is further supported by evaluation method proposed by OpenFace on those AUs, which also have high scores of 0.85 and 0.59. Additionally, optical flow is used to visually compare the original facial movements and the transferred facial movements. With this article, we publish our dataset to enable future research and to increase the data pool of micro-expressions research, especially in the spotting task.

## 1. Introduction

Facial expressions can be divided into two main categories: macro-expression (MaE) and micro-expression (ME). MaE, also known as regular facial expression, generally lasts between 0.5 to 4 s [1] and often has high intensity movements, while ME is subtle, typically lasting less than 0.5 s [1]. ME occurs more frequently in stressful and high-stake situations [2,3]. Due to its involuntary nature, analysing MEs can reveal the emotional state of a person [4].

Deep learning is a powerful but data-hungry pattern recognition technique, which could potentially advance the current state of MaE/ME video recognition tasks. Compared with other mainstream datasets (e.g., facial expression datasets), ME datasets are relatively scarce. In current ME recognition research, there is a serious problem in the extensive amount of false positives [5,6]. In addition, ME and MaE can occur independently in a real-world scenario. Hence, ME spotting is proposed as a preliminary elimination for possible false positives. A long video based ME dataset is needed as it resembles a more realistic situation.

In the progress towards fully automated analysis of MEs and MaEs, recent facial micro-expression challenges referred to ME spotting as an imminent practical first-in-the-pipeline problem [6,7]. As such, long video datasets are key to developing robust spotting algorithms of ME and MaE occurrences, which could further advance the field to unconstrained “in-the-wild” settings. However, to date there are only two related datasets with ME and MaE, which are SAMM Long Videos (SAMM-LV) [8] and CAS(ME)^2^ [9]. Therefore, there is a high motivation to obtain more data.

Facial movements are often labelled using the well-established Facial Action Coding System (FACS) [10]. Labelling is best conducted using at least two certified FACS coders, as high inter-reliability is the key to ensure the accuracy of annotations. The process of FACS coding is time-consuming, therefore, many researchers attempted to automate FACS coding [11,12]. An automated computerised scoring system is efficient but often requires multiple stages, including region of interest detection, segmentation, and classification. Whenever there is a classification error, it is difficult to troubleshoot, which is the main reason why the established datasets were annotated by human FACS coders.

This paper uses a Generative Adversarial Network (GAN) as an advanced image augmentation. Our approach mainly preserves facial motion and changes the identity of the participant to produce new data. This approach is fully automated and able to generate unlimited amount of data without any supervision. This method also has an added benefit of reusing the ground truth labels without the need of additional FACS coding.

The main contributions of this paper are:

1. The first synthetic facial macro- and micro-expression style dataset using style transfer on pre-existing dataset.
2. We study the correlation of the original and synthetic data using AUs detected by OpenFace.
3. We recommend to use optical flow for facial motion transfer analysis, to visualise the facial movements and its intensity.
4. We share our synthetic dataset with the research community and present future challenges in spotting expressions (particularly micro-expressions) on long videos.

## 2. Related Work

One of the main solution to prevent over-fitting in deep learning is to increase the variation of data. It is commonly done by data augmentation. Data augmentation can be classified into two main categories: basic image manipulations and deep learning approach [13]. Examples of basic image manipulations are applying rotation, translation, and adjusting contrast and brightness. Although this method is widely used, over-extensive colour augmentations may cause a deep model to overfit worse than the original [13]. Moreover, Sucholutsky and Schonlau [14] emphasised the importance to increase the number of unique features, but it is unclear if data augmentation helps in this aspect.

Deep learning approaches like neural style transfer can increase data variation. This technique takes the extracted style from an image and maps it to a source image. The origin of this method is based on Gatys et al. [15] using different convolutional layers of a CNN. The results are not realistic. Combination between CNN and Markov random field by Li et al. [16] is an improvement on Gatys et al. [15], as it is able to transfer style onto buildings and cars realistically. Semantic style transfer [17] by obtaining a semantic map using a CNN is another interesting method. For face, non-matching facial features need to self-align using customised semantic maps and the results are coarse compared to the real data.

Face-centred style transfer is demonstrated by Shih et al. [18]. This model uses multiple energy maps which transfer only the colour of the style while retaining most of the facial features, which are well preserved. However, it is very sensitive to lighting, so if the source and reference do not have same lighting, the results will become unrealistic. The generated faces remain visually recognisable from the original images despite the colour changes.

GANs are used primarily in generating realistic artificial data using a zero-sum game by at least two neural networks (at least one generator and one discriminator). In facial expression generation, a few ways to generate new data include generating AUs on a neutral face are GANimation [19] and deepfake-like facial motion transfer by Siarohin et al. [20].

Video generation GANs are also capable of generating facial expressions. Vondrick et al. [21] uses spatiotemporal convolution in both the generator and discriminator. However, this method has an upper limit of approximately 1 s duration in the videos generated as its generator is trained wholly with 32-frame videos. Saito et al. [22] proposed an end-to-end model that uses a temporal generator and an image generator. MoCoGAN [23] generates video by mapping random vector sequences to video frame sequences. It retains the content image feature while the motion part is treated as a stochastic process. These methods are able to generate video sequences with facial expressions, however the generated images are impractically low-resolution (e.g., 64 × 64). MEs are typically very subtle motion, thus excessive down-sampling risks discarding the relevant features.

AU Intensity Controllable Generative Adversarial Network (AU-ICGAN) proposed by Xie et al. [24] is the only ME related facial action unit (AU) transfer. It uses both CASME II [25] and SAMM [26] datasets as reference, and source frames by transferring AU of CASME II onto SAMM participants and vice versa. Nonetheless, this paper did not report any quantitative or qualitative evaluation of the generated data. The generated data are also not publicly available, which is contrary to the justification for synthetic ME data. Additionally, the identity of the generated participants remain the same in all the examples given and as a result, the variety of the participants is limited.

Our findings show there is limited research in generating synthetic long videos for MEs and the quality of the generated data were not properly evaluated. To fill this research need, we propose the use of StarGANv2 [27] to perform reference guided style transfer on ME-based long videos for data generation and recommend evaluation techniques to assess the quality of the generated data.

## 3. Method

**Figure 1:**
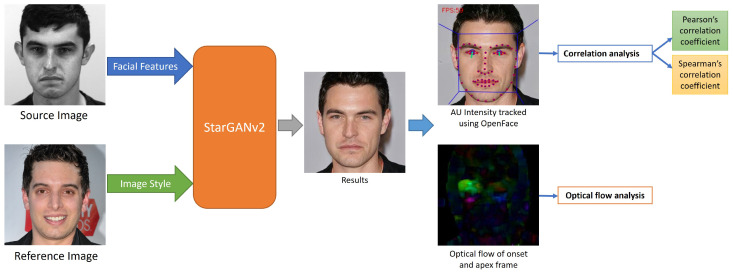
Overall pipeline of our method with analysis. StarGANv2 is used to modify the “style“ of participant from SAMM-LV dataset [8]. The synthetic image exhibit the style extracted from the reference image (from FFHQ dataset [28]) while maintaining the facial features of the source image. Facial Action Units (AUs) of the synthetic image is measured using OpenFace [29]. The AUs and optical flow of the synthetic image are compared with the original source image.

We take advantage of StarGANv2’s [27] ability to extract style from one image to a target image and propose a novel method of transferring ME and MaE movements using style transfer. This is an advanced image augmentation approach, which essentially creates unique face features and preserves facial movements. The overall pipeline of our method (including analysis) is shown in Figure 1.

### 3.1. Datasets

Facial micro- and macro-expressions dataset, SAMM-LV, is used as source frames, which is the target for style transfer. SAMM-LV [8] is an extension of SAMM dataset [26] that has 147 long videos containing 343 MaEs and 159 MEs with frame rate of 200 fps.

The generative algorithm (StarGANv2) uses CelebA dataset [30] as the training set. CelebA is a face-based dataset which contains ten thousand unique identities, where each of them has twenty images. The reference frames (containing 14 female and 14 male adults) are selected from FFHQ dataset [28], another face-based dataset, so that more variety of generated faces can be produced. This can also prevent StarGANv2 from using its training set for style transfer and demonstrate the robustness of style transfer of the model.

### 3.2. Style Transfer

StarGAN [31] is able to perform image-to-image translation for multiple domains using a scalable model. This model demonstrates its effectiveness on facial attribute transfer and facial expression synthesis. StarGANv2 [27] further enhances it by replacing domain labels with domain-specific style codes. Compared to the previous version [31], it has additional structures: a mapping network and a style encoder. The mapping network learns to transform random Gaussian noise into a style code, while the encoder extracts a style code from the reference image. StarGANv2 modifies the style of the source frame containing ME using a reference frame. This is performed by applying a style reconstruction loss (shown in Equation (1)) onto the generator to utilise the style code during the image generation process, referred to as reference-guided image synthesis.

(1) $$L_{sty}=E_{x,y,z}\left[s-E_{y}\left(G\left(x,s\right)\right)\right]$$

where *E* is the encoder, *G* is the generator, *x* is the original image, *y* is the domain, *z* is the latent code and s is the style code.

In our experiment, we use StarGANv2 pre-trained on the CelebA dataset [30] for reference-guided image synthesis.

## 4. Results and Discussion

We generated a synthetic dataset, named SAMM-SYNTH, using reference-guided image synthesis of StarGANv2 on SAMM-LV dataset. To evaluate the quality of the generated dataset, first we perform quantitative analysis using facial action units (AUs) detected by OpenFace. We conduct a correlation analysis on the AUs in SAMM-LV and SAMM-SYNTH. To compare the visual appearances, we perform qualitative analysis that involves the use of optical flow.

### 4.1. Generated Data

**Figure 2:**
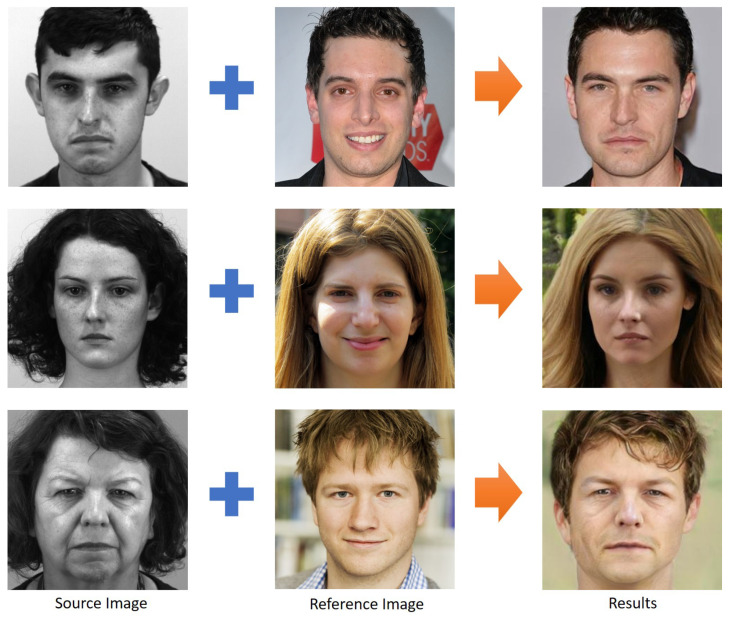
Style transfer using StarGANv2, also known as reference-guided image synthesis. The result exhibits facial features of the source image (taken from SAMM-LV dataset [8]), while taking the style extracted from the reference image (taken from FFHQ dataset [28]). StarGANv2 is capable of transferring style for both gender realistically. Inter-gender style transfer is also possible as shown in the bottom row.

The results of StarGANv2 reference-guided image synthesis can be seen in Figure 2. We observed that the facial attribute follows the source image (taken from SAMM-LV) while the style follows the reference image (taken from FFHQ dataset). We conducted style transfer on each participant of SAMM-LV. As a result, SAMM-SYNTH consists of 147 long videos with 15 female and 15 male participants. We generated most synthetic data by following their original identified gender (other than 1 female participant, due to excessive artifacts). We also found that inter-gender style transfer is possible. An interesting observation is that we found StarGANv2 tends to generalise female participants to have long hair. This may be caused by the training set (CelebA) which consisted of mostly female participants with long hair.

### 4.2. Action Unit Analysis Using OpenFace

OpenFace 2.0 [29] is a facial analysis toolkit that is capable of performing facial landmark detection, head pose estimation, facial AU recognition and eye-gaze estimation. OpenFace uses Convolutional Experts Constrained Local Model (CE-CLM) [32] for facial landmark tracking and detection. CE-CLM uses a deep convolutional neural network for the 84-point facial landmark detection. Based on the facial landmarks, the AU intensity of both the original and synthetic data are measured.

**Figure 3:**
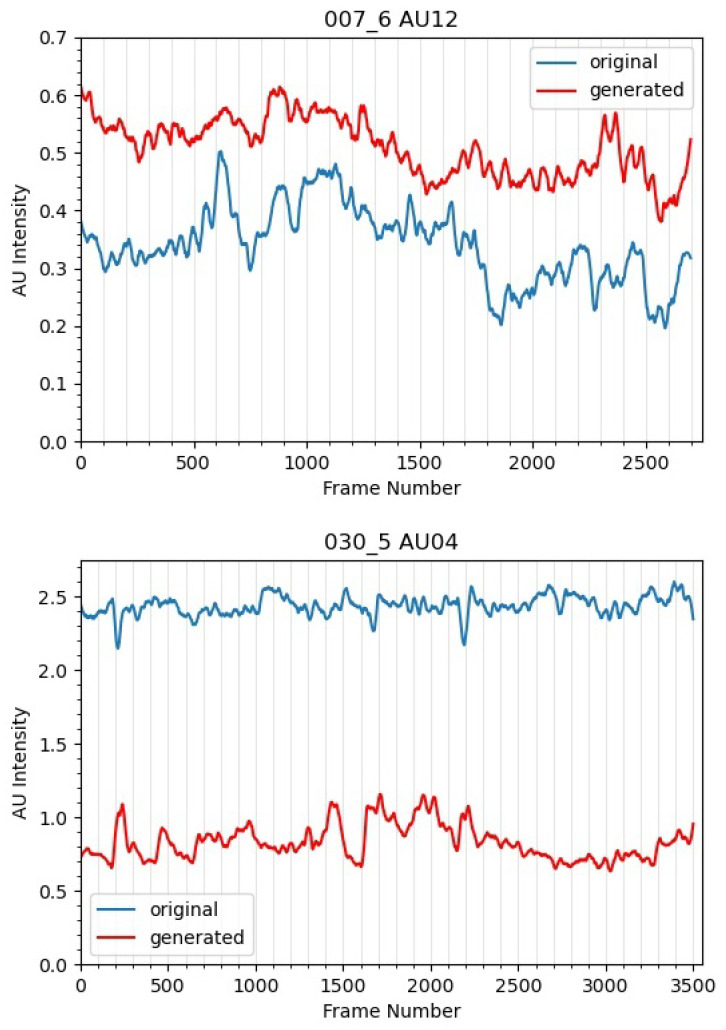
Illustration of AU intensities measured by OpenFace for selected videos of 007_6 and 030_5 of SAMM-LV and SAMM-SYNTH. AU12 is amplified, while AU4 is softened in the synthetic data. According to OpenFace’s documentation, AU intensity has a range of 0 (not present) to 5 (maximum intensity). However, the AU intensity is standardised based on the initial frame. The changes in AU intensities indicate movements of that AU which is what we are interested in.

Two selected AU intensities measured by OpenFace for both original and synthetic videos are shown in Figure 3. They were smoothed using Savitzky-Golay filter [33]. The rise and falls of the AU intensity indicates movement on each particular AU. We observed that the AU12 movement is better replicated compared to AU4. Another reason may be resorted because AU12 (lip corner puller) has a bigger movement range when compared to AU4 (brow lowerer).

We analyse the similarities of facial movements between SAMM-LV (original) and SAMM-SYNTH (transferred) using Pearson’s and Spearman’s correlation [34]. To analyse the quality of AU transferred, each AU intensity (original and synthetic data) measured detected by OpenFace are transformed into Z-score using Fisher’s Z-transformation [35] as in Equation (2). The average values of Z-score are calculated and converted to correlation coefficients (Pearson’s and Spearman’s coefficients). The reason for using Z-score when calculating the average is that correlation coefficients are non-additive. Hence, a sample-size weighing (e.g., Fisher’s Z-transformation) must be applied before averaging.

(2) $$z=\frac{1}{2}ln\left(\frac{1+r}{1-r}\right)=tanh^{-1}\left(r\right)$$

where *z* is Fisher’s Z-score and r is the correlation coefficient.

We tabulate the results sorted by AU and by participant in Table 1 and Table 2, respectively. Table 1 compares the Pearson’s correlation coefficients sorted by AU, we observed that AU45, AU12 and AU6 are better replicated as they show higher Pearson’s correlation coefficients of 0.92, 0.74 and 0.72, respectively. Based on OpenFace benchmark, when compared to the ground truth labelling, OpenFace performs better in AU4, AU12 and AU25 with a Pearson’s coefficient of 0.70, 0.85 and 0.85 respectively, on a facial expression dataset (DISFA dataset [36]). By comparing Pearson’s coefficients of our experiment and OpenFace benchmark, we have a high confidence that AU12 is the best transferred AU. This is proved whereby OpenFace has high accuracy and our experiment shows high correlation in AU12.

**Figure 4:**
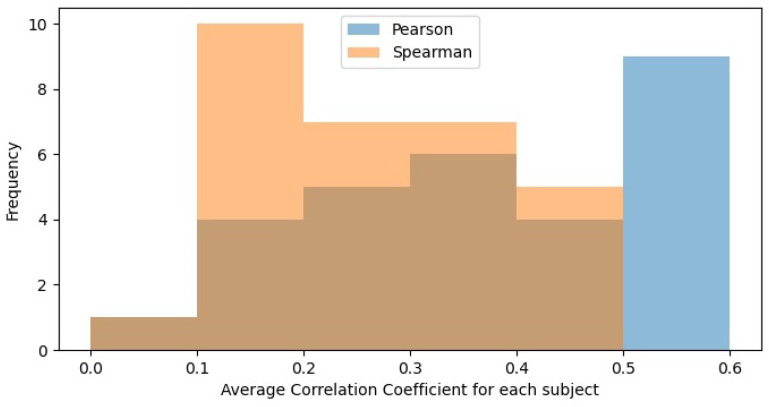
Histogram of correlation coefficients on all participants of SAMM-LV and SAMM-SYNTH. Both histograms use 6 bins. We observed that Spearman’s coefficients are more right-skewed relative to Pearson’s coefficient. This implies that our results are more linear than monotonic correlated.

In Table 2, we observed that the overall mean Pearson’s correlation is higher than Spearman’s correlation. This implies that our data is more linear than monotonically correlated, as Pearson’s correlation measures the linearity of the data correlations while Spearman’s measures the monotony of the data correlations. Figure 4 further confirms our claim as the Spearman’s distribution is skewed right, indicated a lower boundary in the coefficients.

**Figure 5:**
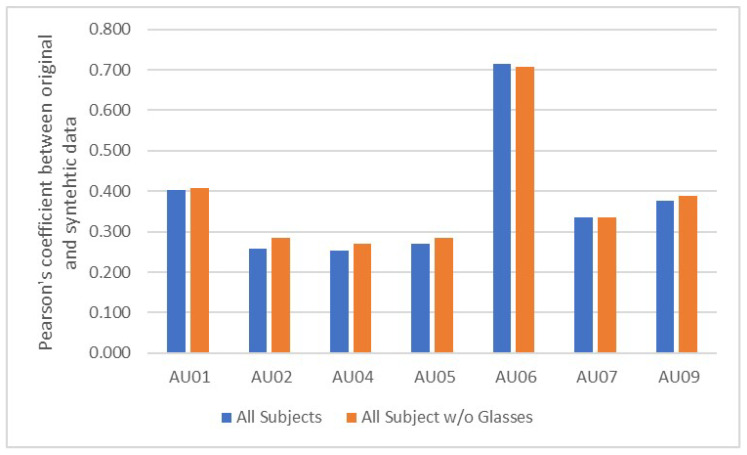
Comparison of Pearson’s correlation of upper face AUs between all participants and those without glasses. By excluding participants with glasses, most of the upper face AUs improved.

We identified participants with glasses (participant 010, 020, 021, 032 and 037) and did a separate comparison by removing them from the whole dataset. In Figure 5, we can see that most of the Pearson’s coefficient improves for the upper face AUs. The only AUs that do not improve are AU6 and AU7. These two AUs can affect the eye corners and muscles around the eye socket. The outer frame of the glasses might exaggerate the movement in the generation process resulting in higher correlation. This presents a challenge for style transfer on participants with glasses.

### 4.3. Optical Flow Analysis

**Figure 6:**
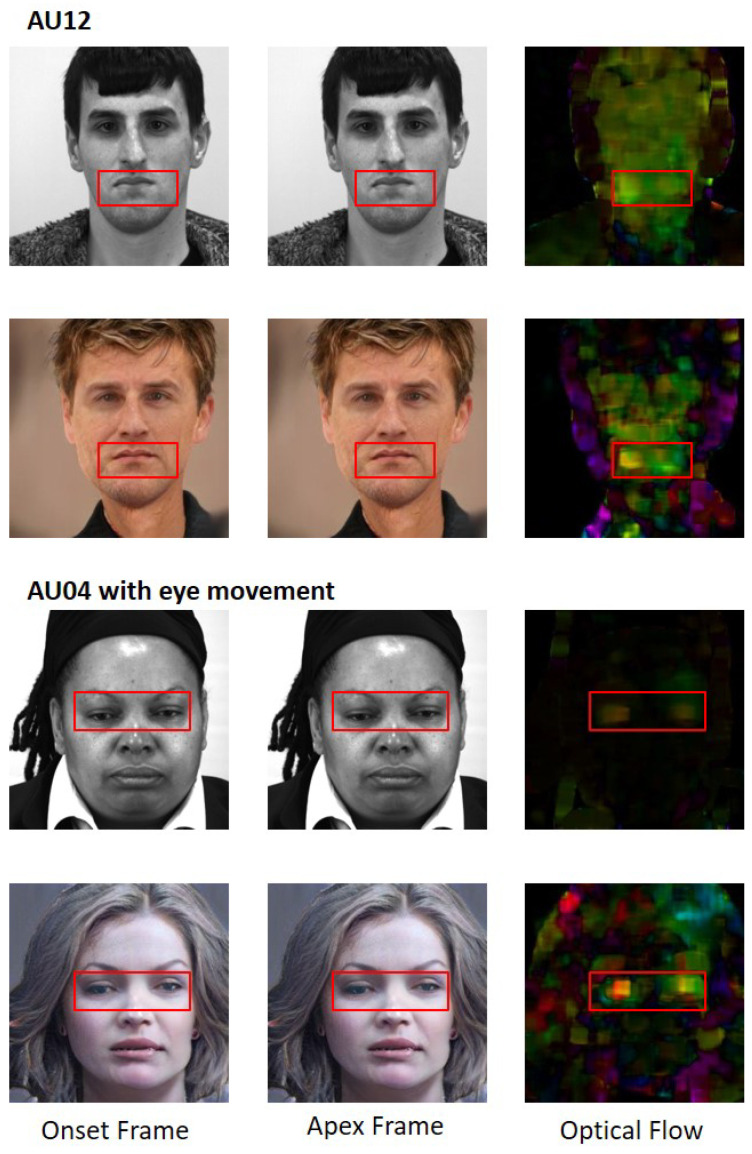
Optical flow comparison of onset and apex frames between original and synthetic MEs. Each respective AUs are shown in the red bounding boxes. (**Top**) For movement 007_6_5, AU12 is involved. (**Bottom**) For movement 030_5_1, AU4 and slight eye movement are involved. The hue represents direction, while the brightness represents intensity of the movements. Note: Original images were scaled to 256 × 256 pixels for fairer evaluation.

We use optical flow to visualise the facial movements and their intensities in both original and synthetic data. Farneback optical flow [37] is used to calculate the frame differences between the onset and apex frame of ME related movements as shown in Figure 6. In image sequences of facial expression, the onset frame is the frame where the expression starts and apex frame is where the expression is at the highest intensity. For the optical flow method, we assume that all participants were captured under uniform lighting at all times. The direction and intensity of the movement is determined by the hue and brightness of the HSV colour model. For comparison, the original images were scaled down to 256 × 256 to match the synthetic images. In AU12 movement, we observed that the right lip corner movements in both original and synthetic pairs are well replicated (bright spot on the optical flow images). In AU4 movement, the eye brow movements are less distinct, while the eye movements are better replicated. In both optical flow of synthetic data, we noticed that additional frame differences are observed around the jaw region, although it is not visible to the human eye, it was captured in the optical flow analysis.

### 4.4. Advantages

The main advantage of our approach is that the generated faces have a new style. This allows a neural network to be trained or validated on unseen data, which can potentially result in better model generalisation. It has an added benefit of protecting the identity of the original participant while generating new data. GAN generated methods [19,23] require substantial labelled data. For our case, due to the limited MEs and MaEs long video datasets, generation could be a challenging task. Style transfer resolves this issue as it primarily transfers the facial movements without the need of training. Moreover, style transfer is a simple and convenient method of data augmentation. By altering the reference frame alone, we are able to create new faces with ME or MaE included in the synthetic video. We can also generate cross-ethnic faces (as participant 030 in Figure 6) to increase the diversity of ME dataset, which primarily consists of participants from one particular country and ethnic background [38]. It is known that demographic imbalance of the training dataset will cause external biases on the trained models [39,40,41] that results in inaccurate and erroneous predictions. With the ability to generate wide range of participants with different demographics, this can alleviate the issue. Potentially, this will help to improve the generalisation of the deep learning models. It is also relatively low in computational cost. By training StarGANv2 once, we are able to generate an unlimited amount of style transfer data with very little computational cost.

### 4.5. Limitations and Challenges

**Figure 7:**
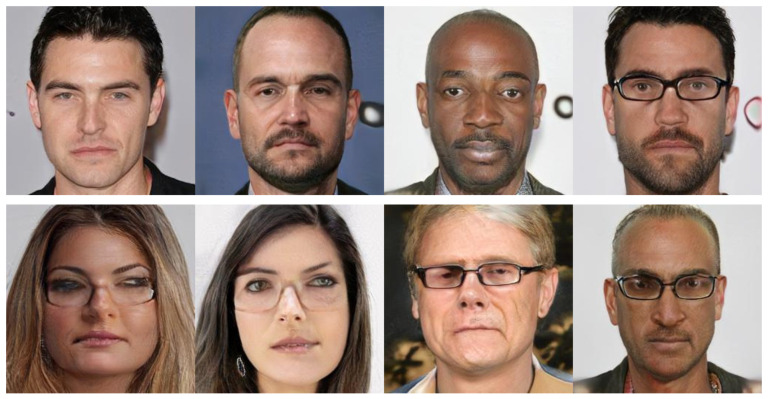
Artifacts present in synthetic images. (**Top**) Background artifacts of blob-like structure. (**Bottom**) Participants with glasses are not realistically generated.

We conduct extensive tests on our synthetic data by changing the reference images for each participant of SAMM-LV. We found that the background of the reference image is one of the source of artifacts. Non-uniform background creates patches of artifacts which are not realistic. Hence, all the reference images are selected with a uniform background. However, there are still blob-like artifacts in some cases (source might originate from the generator that learnt unnatural distributions that can fool the discriminator). Next, source images with facial accessories (e.g., glasses) create unrealistic images. StarGAN treats glasses as a facial feature and attempts to blend it onto the face. Not only are the images are not suitable for real life applications, our separate analysis, which only includes participants without glasses (in Figure 5), shows improvement in the similarity of AUs transferred. This shows that the facial movement of participants with glasses are not well transferred. Facial accessories come in various sizes and shapes, which is challenging to transfer. They are also not well-represented in StarGAN’s training set. The artifacts mentioned are shown in Figure 7. A potential solution is to retrain the model on a new training dataset with facial accessories or inpaint the glasses before the style transfer process.

**Figure 8:**
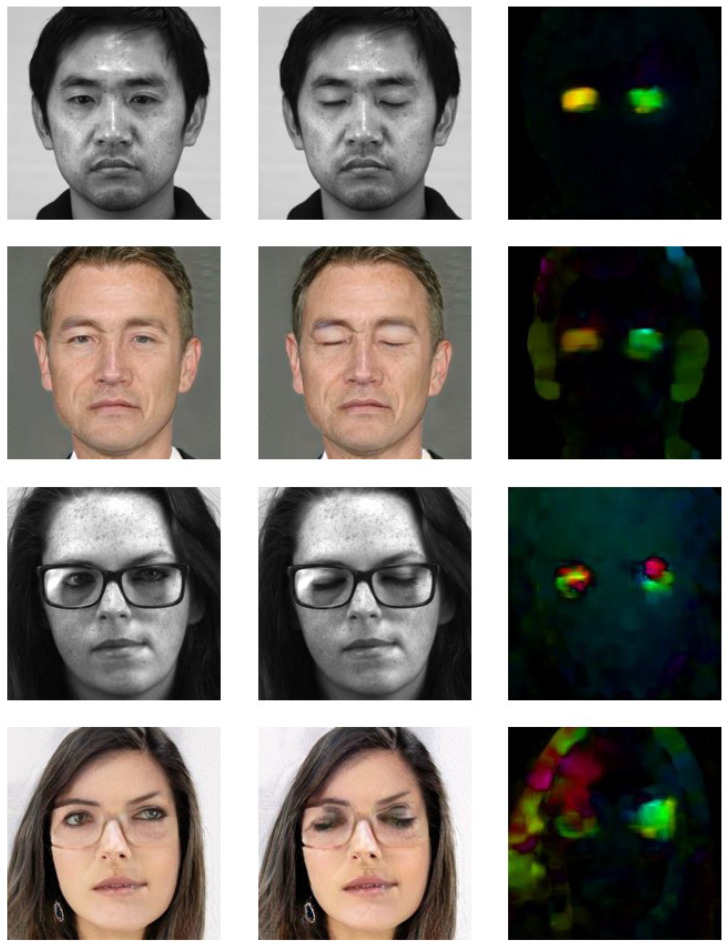
Eye blinks can cause changes in other facial regions. (**Top**) Mild case which results in small changes of hair and ears in the synthetic images. (**Bottom**) Extreme case on participant with glasses which results in huge changes.

We noticed that when eye blinks occur in the source images, the hair structure of the synthetic images was slightly altered. This might be caused by the pre-trained weights. The majority of the face images in CelebA are faces with open eyes, hence, it is reasonable to assume that when a participant blinks, the model wasn’t able to preserve the face structure well, especially the hair. Selected examples with this issue are shown in Figure 8.

All the AUs of original and synthetic data are positively correlated. However, different AUs have different range and sensitivity, which may explain the low correlation coefficients in some AUs. The source images from SAMM-LV do not have a balanced distribution across all AUs, which may result in an uneven comparison that skew the results towards AUs that are more common.

## 5. Conclusions

We showed that style transfer on a pre-existing long video dataset (SAMM-LV) can be used as a method of generating a new synthetic dataset – SAMM-SYNTH. We found that AU6, AU12, and AU45 are AUs that transferred well in the SAMM-SYNTH using Pearson’s correlation. We performed facial motion transfer analysis using optical flow to visualise the movements on both the original and synthetic data. Like in other GAN-based approaches, we observed the synthetic data were affected by visual artifacts.

Future work includes addressing the style transfer issues related to eye blinks, eye glasses, and the visual artifacts. We will expand the training dataset of the style transfer model to include other face datasets and design a new method. To evaluate the effectiveness of synthetic data as a data augmentation technique in this domain, we will add SAMM-SYNTH to the current training pool and conduct ME related experiments to investigate the use of SAMM-SYNTH in ME recognition, and spotting ME and MaE in long videos. We will share SAMM-SYNTH for research purposes upon the acceptance of this paper.

## Figures and Tables

**Table 1:** Analysis of Action Units (AUs) detected by OpenFace on original SAMM-LV participants and SAMM-SYNTH synthetic videos. The bolded values are the top-3 highest Pearson’s correlation coefficients. Pearson’s correlation coefficients show strong positive correlation for AU6, AU12, and AU45, which are well preserved compared to the other AUs. “Benchmark” indicates the Pearson’s coefficients of OpenFace detection versus ground truth labelling on DISFA dataset [36], which reported that OpenFace has better performance on AU4, AU12, and AU25 detection.

Action Unit	SAMM-LV vs. SAMM-SYNTH	Benchmark
AU1	0.40	0.64
AU2	0.26	0.50
AU4	0.25	**0.70**
AU5	0.27	0.67
AU6	**0.72**	0.59
AU7	0.33	-
AU9	0.38	0.54
AU10	0.28	-
AU12	**0.74**	**0.85**
AU14	0.42	-
AU15	0.26	0.39
AU17	0.15	0.49
AU20	0.28	0.22
AU23	0.40	-
AU25	0.26	**0.85**
AU26	0.20	0.67
AU45	**0.92**	-

**Table 2:** Analysis of the quality for each participant of SAMM-LV versus SAMM-SYNTH. Both Pearson’s and Spearman’s correlation are compared. Participant 020 (in bold) ranked the highest, while Participant 024 ranked the lowest.

Participant	Pearson	Spearman
006	0.58	0.44
007	0.38	0.32
008	0.33	0.11
009	0.22	0.13
010	0.38	0.27
011	0.57	0.44
012	0.59	0.38
013	0.25	0.17
014	0.49	0.32
015	0.60	0.41
016	0.19	0.14
017	0.13	0.13
018	0.51	0.40
019	0.40	0.25
**020**	**0.61**	**0.47**
021	0.20	0.15
022	0.41	0.28
023	0.44	0.37
024	0.04	0.05
025	0.51	0.39
026	0.55	0.27
028	0.56	0.27
030	0.27	0.14
031	0.16	0.17
032	0.22	0.15
033	0.51	0.35
034	0.36	0.21
035	0.33	0.25
036	0.39	0.31
037	0.19	0.16
**Mean**	0.39	0.27
**Standard Deviation**	0.19	0.12

## Data Availability

Publicly available datasets were analyzed in this study. This data can be found here: http://www2.docm.mmu.ac.uk/STAFF/M.Yap/dataset.php, accessed on 1 August 2021.
